# Assessment of Various Nanoprimings for Boosting Pea Germination and Early Growth in Both Optimal and Drought-Stressed Environments

**DOI:** 10.3390/plants13111547

**Published:** 2024-06-03

**Authors:** Gordana Tamindžić, Sergei Azizbekian, Dragana Miljaković, Maja Ignjatov, Zorica Nikolić, Dragana Budakov, Sanja Vasiljević, Mila Grahovac

**Affiliations:** 1 Institute of Field and Vegetable Crops, National Institute of the Republic of Serbia, 21000 Novi Sad, Serbia; dragana.bjelic@ifvcns.ns.ac.rs (D.M.); maja.ignjatov@ifvcns.ns.ac.rs (M.I.); zorica.nikolic@ifvcns.ns.ac.rs (Z.N.); sanja.vasiljevic@ifvcns.ns.ac.rs (S.V.); 2 Institute of Physical Organic Chemistry, National Academy of Sciences of Belarus, 220072 Minsk, Belarus; s.az@mail.ru; 3 Faculty of Agriculture, University of Novi Sad, 21000 Novi Sad, Serbia; dragana.budakov@polj.edu.rs (D.B.); mila.grahovac@polj.edu.rs (M.G.)

**Keywords:** *Pisum sativum* L., nanopriming, seed germination, growth, drought stress

## Abstract

One of the main climate change-related variables limiting agricultural productivity that ultimately leads to food insecurity appears to be drought. With the use of a recently discovered nanopriming technology, seeds can endure various abiotic challenges. To improve seed quality and initial growth of 8-day-old field pea seedlings (cv. NS Junior) under optimal and artificial drought (PEG-induced) laboratory conditions, this study aimed to assess the efficacy of priming with three different nanomaterials: Nanoplant Ultra (Co, Mn, Cu, Fe, Zn, Mo, and Se), Nanoplant Ca-Si (Ca, Si, B, and Fe), and Nanoplant Sulfur (S). The findings indicate that nanopriming seed treatments have a positive impact on seed quality indicators, early plant growth, and drought resilience in field pea plants established in both optimal and drought-stressed conditions. Nevertheless, all treatments showed a positive effect, but their modes of action varied. Nanoplant Ultra proved to be the most effective under optimal conditions, whereas Nanoplant Ca-Si and Nanoplant Sulfur were the most efficient under drought stress. After a field evaluation, the examined comprehensive nanomaterials may be utilized as priming agents for pea seed priming to boost seed germination, initial plant growth, and crop productivity under various environmental conditions.

## 1. Introduction

Abiotic stressor drought seriously impedes crop production and plants are seriously affected by drought during seed germination and initial seedling growth. Heat and drought seem to be the most important factors restricting crop productivity related to climate change, eventually resulting in food insecurity. Globally, drought conditions are becoming more frequent due to altered patterns of precipitation and decreased rainfall [1,2]. In the last few decades, drought has led to serious reductions in the yields of many plant species, ranging from 30% to 90%, depending on the crop [3]. Additionally, drought impacts plant development and production, affecting mineral nutrition and nutrient density as a secondary effect. Both nutrient uptake and transport are dependent on soil moisture since drought causes a reduction in the transpiration rate and a disturbance in membrane permeability and active transport [4]. The severity of the consequences of drought depends on the plant stage in which it appears as well as the duration of the drought. Due to decreased water imbibition, drought stress lowers seedling vigor and hinders germination, which is the most susceptible stage of plant life [5,6]. Moreover, drought conditions have an impact on plant growth by altering the relationships with water-soluble nutrients, which in turn affects the photosynthetic process and thus ultimately leads to a significant reduction in crop yield [2,7,8]. Moreover, oxidative stress caused by drought harms biological membranes and macromolecules, including DNA, proteins, lipids, and pigments involved in photosynthetic processes [9]. Plants have evolved different mechanisms of adaptation to drought, such as morpho-physiological and molecular changes.

Legume crops such as peas play an important role in agricultural conservation systems and contribute to food security in the developing world, but recently, in many regions of the world, their production has been seriously threatened by drought [10]. With 12.4 million tons of dry peas and 20.5 million tons of green peas produced in 2021 [11], pea production is currently classified as the third main pulse crop globally, after common bean and chickpea, suggesting that peas are an essential resource for food and feed [12]. This is supported by the fact that plant-based protein production is imperative in sustainable agriculture. Peas are a very important species because they provide proteins, fibers, folate, iron, potassium, vitamins, and polyphenols for a healthy diet [13]. It also exhibits multiple health benefits, such as anti-inflammatory effects, through antioxidants, antimicrobials, and regulation of metabolic processes [13]. In addition, peas have the ability to fix atmospheric nitrogen through a symbiotic partnership with rhizobia. The capacity of peas to fix dinitrogen is approximately 165 kg N ha^−1^, although it typically ranges from 40 to 60 kg ha^−1^ [14]. Thus, peas enrich the soil with nitrogen and reduce the use of fertilizers, providing environmental benefits and ensuring a high-quality grain yield rich in proteins as an important target, which is the goal of the European Union Green Deal Farm to Fork strategy [15]. However, as cool-season crops, peas are sensitive to drought in the initial stages of growth and development.

Depending on the frequency and severity of drought stress, drought has various impacts on a crop’s seed germination, vegetative and reproductive growth, and maturity stages [16]. In recent times, numerous strategies have been evaluated to combat drought stress [17]. One useful strategy for managing various abiotic stressors, such as drought, is seed priming. Seed priming is a pre-sowing seed treatment that falls under the category of seed improvement treatments. It permits controlled hydration of seeds so they may imbibe water and go through the initial stage of germination, but it prevents radical protrusion through the seed coat [18]. Seed priming creates a specific physiological state known as the primed state, which enhances numerous cellular responses. Plants are then ready to respond quickly to additional exposure to stress [19,20,21]. Improvements in seed quality have been demonstrated for numerous seed priming methods, including hydropriming, osmopriming, halopriming, biopriming, and hormopriming [22,23,24]. Moreover, it has been reported that seed priming techniques hasten the germination process and enhance seedling emergence rates through changes at the cellular level such as cell division, synthesis of nucleic acids and proteins, accumulation of essential lipids, production of antioxidants, and activation of DNA repair mechanisms [22]. Besides the fact that seed priming techniques boost seed germination in optimal conditions, it is also known that it is possible to develop stress memory in plants through priming strategies, which hastens the molecular responses related to stress tolerance upon subsequent stress exposure [25]. Through increased accumulation of compatible solutes like protective enzymes, amino acids, sugars, proline, and glycine betaine, seed priming techniques lead to effective osmotic regulation that helps maintain better water status in plants and reduce membrane damage in drought stress, ensuring plants’ resistance to drought stress [25,26].

The use of nanoparticles (NPs) in agriculture has gained increasing interest in recent years. New nanoscale (1–100 nm) materials with enhanced biological activity, together with unique and beneficial physical properties derived from their high surface-to-volume ratios, have enabled nanotechnology, a new era of agricultural technology [27]. The goal of using nanoparticles in agriculture and natural ecosystems is to increase the performance and sustainability of plants and soil by using fewer inputs [28]. Various metal-oxide and carbon-based NPs proved to be beneficial regarding seed germination enhancement and initial plant growth under both optimal conditions and abiotic stresses [28,29,30]. Research on the effects of single nanoparticles on the quality of seeds and the early plant growth of different crops is extensive. Nevertheless, the impact of comprehensive nanoparticles on seed quality performance and early plant growth under both optimal and stressful conditions has been the subject of very few studies. Nanoplant Ultra was chosen since it contains NPs of primary, essential elements classified as micronutrients, including iron (Fe), zinc (Zn), manganese (Mn), molybdenum (Mo), copper (Cu), and selenium (Se). These micronutrients are proven to have a vital role in various metabolic processes and are essential for maintaining the normal functioning of many physiological processes [31], while cobalt (Co) is known to have an important role in plant growth and stress resilience in leguminous plants [32]. Nanoplant Ca-Si contains calcium (Ca), silicon (Si), boron (B), and Fe, which are beneficial for germination and initial plant growth. Boron is proven to have a vital role in the metabolism of nucleic acid, carbohydrates, proteins, and indole acetic acid, cell wall synthesis, membrane integrity and function, and phenol metabolism [31]. As well as Si, Calcium plays a key role in reducing abiotic stressors and boosting plants’ internal defenses [33,34]. It has been demonstrated that Ca enhances germination, biomass, and antioxidant defense in plants [33], while Fe is a crucial micronutrient for several biological processes in plant systems, including those relating to respiration, photosynthesis, the quality of plant products, and cellular enzymes in organelles [35]. The main component of Nanoplant Sulfur is sulfur, an essential macronutrient that plays an important role in various cellular metabolic processes [36]. Additionally, seed quality assessment is closely related to the sulfur content of the seeds [36]. Increasing sulfur content through seed priming could be of great importance for pea production. These NPs are mainly used for foliar treatment of crops and have not been studied in the pre-sowing preparation of pea seeds. Plants need a wide range of microelements, so it is necessary to study and use different brands of nanomaterials through novel application methods. Therefore, this study aimed to examine the impact of seed priming with three different comprehensive nanomaterials, namely Nanoplant Ultra (Co, Mn, Cu, Fe, Zn, Mo, and Se NPs), Nanoplant Ca-Si (Ca, Si, B, and Fe NPs), and Nanoplant Sulfur (S NPs), on seed quality performance, initial plant growth, and drought tolerance of field pea under both optimal and drought-stressed conditions.

## 2. Results

The seed quality performance of field peas was estimated to evaluate the effects of nanopriming treatments in optimal and drought-stressed conditions. The germination test was performed in order to evaluate the effects of three nanopriming treatments (Nanoplant Ultra, Nanoplant Ca-Si, and Nanoplant S) on pea seed quality and the initial growth of 8-day-old seedlings under optimal and PEG-induced drought conditions. The obtained results revealed a significant effect of treatments, conditions (optimal conditions and drought), and their interaction on seed germination, initial plant growth, and drought tolerance indexes (Table 1). The condition had a significant effect on all examined parameters of field pea, except on root length stress tolerance index (RLSI) ( p≥0.05). Also, it is evidenced that treatments had a significant effect on seed germination, growth-related parameters, and stress tolerance indexes (Table 1). Furthermore, condition × treatment interaction significantly altered all examined parameters of field pea (Table 1).

**Figure 1:**
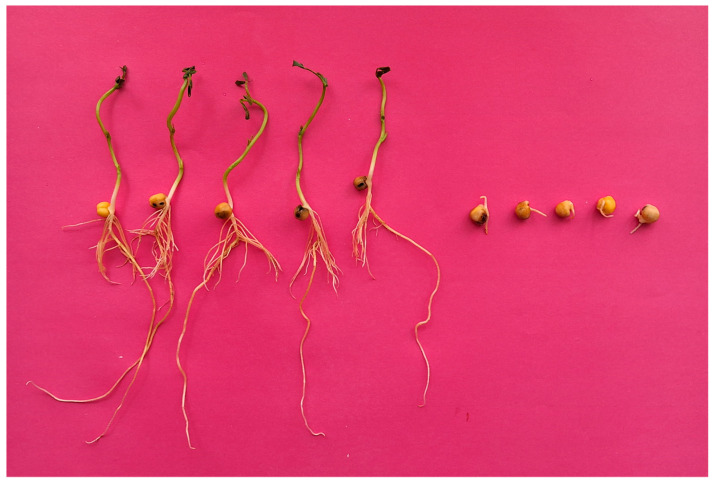
Normal (**left**) and abnormal (**right**) seedlings of 8-day-old field pea.

In general, the parameters of field pea increased significantly as a result of the examined nanopriming treatments under optimal conditions and drought (Table 2, Table 3 and Table 4). Table 2 demonstrates the effect of the examined nanopriming treatments on field pea germination (first count on the 5th day) and final germination (final count on the 8th day), as well as seedling length (on the 8th day) under optimal conditions (Table 2a) and artificial drought (Table 2b). On average, all examined priming treatments altered germination (first count) in comparison to the control under optimal conditions. The highest increase in germination (first count) was observed in priming with NP Sulfur (+8.5%), followed by priming with NP Ca-Si (+7.5%) as compared to the control. Priming with NP Ultra also significantly increased the germination (first count) of field peas, but to a lesser extent. Also, a significant effect of all nanopriming treatments in relation to the control was observed in final germination, shoot length, and root length (Table 2a). The highest increase in final germination over control was observed after nanopriming with NP Ca-Si, followed by NP Ultra and NP sulfur (+8.0%, +5.8%, and +3.3%, respectively) in comparison to the control. The effect of nanopriming treatments was also determined by the percentage of abnormal seedlings (%) that do not develop into satisfactory plants with a well-developed root and shoot system (Figure 1). A significant decrease in the percentage of abnormal seedlings was observed in all examined priming treatments, except in the NP Ca-Si treatment, where the decrease in abnormal seedlings was not significant. Moreover, the shoot length of pea seedlings was significantly improved by all examined nanoprimings as compared to the control and hydropriming; NP Ca-Si led to the highest increase in shoot length in comparison to the control, followed by NP Ultra and NP Sulfur (+24.6%, +18.0%, and +6.8%, respectively). However, only nanopriming with NP Ca-Si followed by nanopriming with NP Sulfur led to a significant increase in root length as compared to the control (+10.5% and +7.5%, respectively) and other priming treatments.

Drought considerably decreased field pea final germination and initial growth (Table 2b). Under drought-stressed conditions, the effects of the examined priming treatments had a slightly different pattern on germination and shoot length. Namely, hydropriming had the greatest effect on germination (first count) (+21.0%), followed by NP Ultra (+13.6%) and NP Ca-Si (+12.3%). The highest final germination was observed in priming treatment with NP Sulfur, followed by NP Ca-Si and NP Ultra (+11.3%, +8.5%, and +6.5%, respectively), while hydropriming had no significant effect on final germination. However, no significant effect of the examined priming treatments on abnormal seedlings was observed. Furthermore, all nanopriming treatments resulted in a notable increase in shoot and root length compared to the control under drought (Table 2b). Priming with NP Ultra stood out as the most effective, as it increased shoot length by +25.4% and root length by +8.1% compared to the control. Other priming treatments also had a positive effect on shoot and root lengths in drought-stressed conditions but to a lesser extent.

The effect of nanopriming treatments (Nanoplant Ultra, Nanoplant Ca-Si, and Nanoplant Sulfur) on the fresh and dry weight of 8-day-old pea seedlings in both optimal and PEG-induced drought conditions is presented in Table 3a,b, respectively. On average, all nanopriming treatments positively affected field pea fresh and dry shoot biomass accumulation compared to the control (Table 3a). The NP Ca-Si treatment stood out as the most effective in terms of fresh shoot weight, dry shoot weight, and dry root weight (+20.4%, +15.6%, and +63.3%, respectively) compared to the control under optimal conditions. Regarding fresh root weight, NP Sulfur priming treatment had the greatest effect (+38.2%), followed by NP Ca-Si (+28.9%), while NP Ultra had no significant effect on this parameter under optimal conditions. Moreover, under optimal conditions, NP Ca-Si and NP Sulfur priming treatments led to the highest increase in dry shoot weight compared to the control and other treatments. In addition to the NP Ca-Si treatment, a significant increase in root dry weight was also recorded in the priming treatment with NP Ultra compared to the control.

Drought-stressed conditions considerably affected fresh and dry shoots and root weight in control (Table 3b). However, the examined nanopriming treatments significantly altered biomass accumulation. Among all the examined priming treatments, NP Ultra stood out as the most effective since it has the greatest effect on fresh and dry shoots and root weight in comparison to the control. Additionally, the accumulation of field pea biomass under drought was significantly improved by other studied nanopriming treatments, namely NP Ca-Si and NP Sulphur, albeit to a lesser extent. The only exception was NP Ca-Si priming treatment in dry shoot weight, where the increase was not significant.

The effects of seed nanopriming treatments (Nanoplant Ultra, Nanoplant Ca-Si, and Nanoplant Sulfur) on shoot and root elongation rate, seedling vigor index, and shoot and root length stress tolerance indexes of the 8-day-old pea seedlings germinated under optimal and artificially induced drought are presented in Table 4a,b. The examined priming treatments significantly affected shoot and root elongation rate, seedling vigor index, and shoot and root length stress tolerance indexes, both in optimal and drought-stressed conditions (Table 4). Under optimal conditions, the shoot elongation rate was significantly improved by the tested nanopriming treatments, while the best effect was achieved by priming treatments with NP Ca-Si (+27.2%) and NP Ultra (+26.9%) compared to the control and hydropriming (Table 4a). Contrary to this, the root elongation rate was affected by the NP Ultra priming treatment (−22.3%), while other priming treatments had no significant effect on this parameter. Additionally, the seedling vigor index was significantly altered due to priming with NP Ca-Si (+24.7%), followed by priming with NP Sulfur (+9.5%) in comparison to the control. Furthermore, under optimal conditions, the highest values of shoot length stress tolerance index (SLSI) were observed in the NP Ca-Si priming treatment, followed by NP Ultra, while for root length stress tolerance index (RLSI), it was in the priming treatment with NP Ca-Si, followed by NP sulfur.

As with the previously studied parameters, the drought affected the reduction in the shoot and root elongation rate and seedling vigor index (Table 4b). Regarding the shoot elongation rate, priming treatment with NP Ultra (+29.9%) had the greatest effect, followed by hydropriming (+19.9%) compared to the control. In contrast, the root elongation rate did not differ significantly across the tested treatments. On the other hand, priming with NP Ultra has been shown to be the most effective in terms of seedling vigor index, SLSI, and RLSI since the highest values were observed under drought-stressed conditions. Other priming treatments also significantly improved these parameters compared to the control but to a lesser extent.

Furthermore, the association between priming treatments (Nanoplant Ultra, Nanoplant Ca-Si, and Nanoplant Sulfur) and different conditions (optimal and artificial drought) is illustrated by the correlation analysis (Table 5). Correlation analysis confirmed the positive effects of nanopriming treatments on final germination and initial plant growth under optimal (Table 5a) and drought-stressed conditions (Table 5b). Overall, under optimal conditions (Table 5a), a positive interrelationship was established between germination (first count) and final germination root length, fresh and dry shoot weight, fresh root weight, seedling vigor index, and RLSI. Final germination was significantly correlated with shoot length, root length, fresh shoot weight, dry shoot weight, dry root weight, shoot elongation rate, seedling vigor index, SLSI, and RLSI. Furthermore, a significant dependence was observed between shoot and root length, fresh and dry shoot weight, dry root weight, shoot elongation rate, seedling vigor index, SLSI, and RLSI. Moreover, dry shoot weight was significantly correlated with all examined parameters, except for abnormal seedlings and root elongation rate, while dry root weight was significantly correlated with fresh and dry seedling weight, shoot elongation rate, seedling vigor index, SLSI, and RLSI. However, no significant correlation was observed for root elongation rate or other tested parameters.

In drought-stressed conditions, germination (first count) was significantly correlated with final germination, root length, fresh and dry shoot weight, fresh root weight, seedling vigor index, and root length stress tolerance index (Table 5b). Moreover, a positive interrelationship was established between final germination and dry root weight, seedling vigor index, shoot length and root length, fresh shoot weight, fresh root weight, shoot elongation rate, and root length, fresh shoot and root weight, dry shoot and root weight shoot elongation rate, and seedling vigor index. Additionally, SLSI and RLSI were significantly correlated with shoot and root length, fresh shoot and root weight, and shoot elongation rate, while RLSI was also significantly correlated with dry shoot and root weight, seedling vigor index, and SLSI. As in optimal conditions, abnormal seedlings and root elongation rate did not significantly interrelate with the other examined parameters.

## 3. Discussion

Vital plant processes such as seed germination and initial plant growth are impacted by drought in multiple ways at different scales, contingent upon the stress frequency and duration [16,17]. The present study revealed that the presence of drought stress had a discernible adverse impact on final germination and germination-related parameters, as well as on plant growth, growth-related parameters, and biomass accumulation at the seedling stage of field peas. The harmful impact of drought stress is reflected in various changes in morphological attributes such as shoot length, coleoptile development, root length, root diameter, root density, and biomass accumulation [17,37,38,39]. Moreover, the detrimental effects of drought stress are evident in numerous changes in physiological characteristics such as chlorophyll fluorescence, chlorophyll a and b content, carotenoids, seedling water content, evapotranspiration, photosynthetic activity, biochemical characteristics such as malondialdehyde, free proline, total phenols, DPPH radical scavenging activity, amylase, protease, and lipase activities, total flavonoids content, and molecular characteristics (stress proteins, aquaporins, and dehydrins), which overall lead to a reduction in seedling growth [16,17,40,41]. All of these changes contribute to a decrease in the growth of seedlings. Deleterious effects of drought at the germination and seedling stages were observed in peas, maize, faba beans, wheat, etc. [42,43,44,45,46], which corroborates with the results of this study. It has been demonstrated that drought-stressed conditions significantly suppressed seed germination and germination-related parameters of peas in the control. Moreover, pea seedlings were affected by drought, which was observed in shoot and root growth as well as fresh and dry biomass accumulation. These findings align with prior research on peas [47,48]. Also, it was observed that drought conditions led to a decrease in shoot and root elongation rate as well as seedling vigor index in the control. In this regard, similar results were also obtained for rapeseed [49]. The detrimental effects of drought might be linked to decreased cell elongation and division, which in turn results in decreased plant growth, as well as decreased water intake from the rhizosphere and transport via the xylem and phloem tissues of pea plants [42]. Moreover, water uptake and use by plants, especially at critical stages of growth, highly determine physiological processes such as transpiration rate, photosynthesis, turgor, growth, and subsequent crop productivity [50]. Water is a key regulator of element cycling in the biosphere, acting as a medium, reactant, or catalyst for most biogeochemical processes, while this conditioning is even more pronounced in water-limited conditions [50].

The promised tool for alleviating the harmful effects of drought is seed priming, a seed enhancement technique that fosters seed germination and enhances initial plant growth and development. Also, nowadays, seed nanopriming has proven to be a promising technique for boosting plant growth and stress tolerance [30]. Because of their small size and unique physiochemical characteristics, nanoparticles can change seed metabolism and hormonal balance, improve catalysis, transfer required materials, adsorb substances of interest, and increase resistance to environmental stressors [28,30,51,52]. Since NPs can enter seeds through nanoscale holes in the seed coat and deposit on embryonic tissues, seed nanopriming has several advantages over salts [53,54,55]. Moreover, Dimkpa et al. [56] found that both NPs and their salts positively affected crop growth, yield, and grain quality of soybeans under drought stress in greenhouses, indicating that ion release from the NPs triggers their reactivity.

By supplying nutritional components, promoting antioxidant activity, or initiating defense responses, we could alter seed physiology [30]. Studies using colloidal, aggregated nanoparticles like those in our research are uncommon, although a variety of NPs have been studied as priming agents [27,28]. The results of our study showed that nanopriming had positive effects on pea final germination in both optimal and drought-stressed conditions. Priming with NP Ca-Si proved to be the most effective in terms of final germination under optimal conditions, with an increase in final germination of +8.0%, while priming with NP Sulfur proved to be the most effective under drought, with an increase in final germination of +11.3%. In this regard, Si-based nanomaterials, such as silicon dioxide NMs (nSiO_2_) and silicon carbide NMs (nSiC), have demonstrated efficacy in enhancing rice seed germination under optimal conditions [57], as well as marigold and wheat seed germination under optimal and drought-stressed conditions [58,59]. Moreover, CaO NPs have proved to be effective in enhancing seed germination of canola and carom under drought stress since polyamines are produced as a result of elevated Ca^2+^ levels, and these compounds act as growth promoters and enhance seed germination [33,60]. However, there are statements that Ca NPs can be detrimental to seed germination in concentrations higher than 40 ppm [61]. Like other essential macronutrients, sulfur regulates the metabolism of seeds involving proteins, carbohydrates, and oils. On the other hand, S metabolism in seeds has great significance regarding S-containing seed storage proteins, which are the important resources of N, C, and S required for seed germination [36]. Additionally, seed germination is influenced by different S-regulated functional proteins and phytohormones stored in seeds [36]. Moreover, S is essential for the regulation of its translocation in seeds via sulfate transporters. Sulfate mostly passes to the embryo and is used for cysteine biosynthesis and incorporatation into proteins, or it enters the seed coat and endosperm and is utilized for the biosynthesis of defense-related S compounds [36]. Regarding sulfur NPs, these results can be explained by the fact that sulfur regulates seed quality and numerous cellular metabolic processes and that sulfur compounds, such as the tripeptide glutathione (GSH), are involved in the detoxification of reactive oxygen species (ROS) in response to different abiotic and biotic stresses [62,63,64,65]. Also, it has been demonstrated that sulfur significantly stimulated catalase (CAT) and superoxide dismutase (SOD) activities and enhanced the antioxidant compounds under stressful conditions [66]. Moreover, priming with NP Ultra also had a significant favorable effect on germination (first count) and final germination of field peas under both conditions, but to a slightly lesser extent, while it was observed to have a significant effect on the reduction in abnormal seedlings in optimal conditions. The positive effects of NP Ultra were also observed in various pea genotypes [23]. All tested priming treatments did not affect abnormal seedlings in drought-stressed conditions.

Regarding plant growth, shoot length was significantly enhanced by priming treatments with NPs. In optimal conditions, the highest increase was observed in NP Ca-Si treatment, followed by NP Ultra and NP Sulfur, while in drought-stressed conditions, NP Ultra, followed by NP Ca-Si and NP Sulfur, had the greatest effect on shoot length. Also, root length was increased due to priming with NP Ca-Si, followed by NP Sulfur in optimal conditions, and NP Ultra, followed by NP Sulfur and NP Ca-Si in drought conditions. The beneficial effects of priming with NP Ca-Si on plant growth can be attributed to the necessity of calcium for the creation and maintenance of lamellary systems in cell organelles, which may be sufficient to explain why calcium is essential for meristematic growth [67]. Due to their localization in cell walls, Ca and B have been referred to as apoplastic elements. Nevertheless, these two elements greatly aid in maintaining the integrity of cell walls by binding to pectic polysaccharides. It has been demonstrated that B binds to the mamnogalacturonan II (RG-II) regions in conjunction with Ca to maintain CDTA-soluble pectic polysaccharides in cell walls [67]. Moreover, the Ca ion (Ca^2+^) has a multifaceted role as an inter- and intracellular signaling transducer in numerous pathways. These signals are deciphered by various Ca^2+^-binding proteins and corresponding targets, which mediate their transformation into a proper response [68]. The fluctuations in intracellular Ca^2+^ levels regulate various plant growth and developmental processes and mediate the plant response to environmental stimuli, including different stress factors [68]. Also, the other components of NP Ca-Si, Si-based NPs [69,70], B-based NPs [71], and Fe-based NPs [72,73] have all been reported to have favorable effects on the growth of different plant species. It is known that Si NPs lead to different physical, biochemical, and molecular changes in plants and improve tolerance to abiotic (e.g., salinity, drought, heavy metals) and biotic stresses (e.g., pathogens, pests) [74,75]. Si NPs increase seed germination and seed metabolic activity during seedling emergence as well as plant growth and development by increasing gas exchange, photosynthetic rate, transpiration rate, stomatal electrical phenomenon, effective photochemical potency, photosystem II activity, and electron transport rate [76]. Significant enhancements in seed germination and other germination-related parameters could be attributed to the effect of B NPs. Boron is an essential micronutrient that functions in cell-wall synthesis, photosynthesis, transpiration, cell division, cell elongation, nitrogen fixation, tissue vegetative growth and differentiation, etc. [77]. The primary role of boron in plant metabolism is the stabilization of molecules with *cis*-diol groups within complex compounds with glycolipids and glycoproteins present in the plasma membrane, maintaining its integrity and structure [77]. It is also involved in various enzyme reactions and the transport of ions, metabolites, hormones, sugars, and other compounds across the membrane [77]. Iron is a key component of general plant metabolism, including respiration, enzyme activation, DNA and RNA synthesis, energy transfer, photosynthesis, chlorophyll formation, lignin formation, nitrogen reduction, nitrogen fixation, etc. Iron-oxide nanoparticles are preferable among NPs because they are non-toxic, redox-active, magnetically sensitive, and biocompatible [78]. They are easily adhered to the seed coats and are also included in the mobilization of endospermic starch, promotion of seed germination, seedling parameters, root proliferation, and overall plant performance [78]. Furthermore, since nanoparticles are the main actors in plant morphology, growth, and physiology, they can modify leaf protein, alter chlorophyll and total phenolic content (TPC), as well as change the formation of ROS, peroxidase, SOD, CAT, and other enzymes. This explains why peas primed with NP Ultra in drought-stressed conditions had greater shoots and roots [23,42,79,80]. The positive effect on plant growth of peas in drought-stressed conditions was also observed for FeO NPs [79] and MoO_3_ NPs [80]. Molybdenum is a cofactor of several enzymes, including nitrate reductase, nitrogenase, dehydrogenase, and sulfite reductase, where it has structural and catalytical functions and is directly included in nitrogen metabolism and redox reactions [81].

According to Tavanti et al. [53], plants that acquire micronutrient treatments are more resilient to abiotic stress since treatments with low concentrations of B, Cu, Fe, Mn, Mo, Ni, Se, and Zn can stimulate and elicit antioxidative enzymes, non-oxidizing metabolism, and sugar metabolism to reduce the harm caused by oxidative stress. Most of the functions of Cu in plant metabolism are based on its involvement of copper-containing enzymes in redox reactions, while Mn is an important component of detoxifying enzymes and a manganese-containing enzyme complex attached to photosystem II [81]. Selenium affects different physio-biochemical processes, improving the efficiency of photosynthesis, the antioxidant system, and photosynthesis under stress [82]. The beneficial effect of Zn NPs could be related to their role in functional (catalytic) and structural enzyme reactions. Zinc is a component of numerous enzymes and is involved in protein synthesis, carbohydrate metabolism, tryptophan and indoleacetic acid synthesis, membrane integrity, etc. [81]. Furthermore, in terms of biomass accumulation, priming with NP Ca-Si was shown to be the most effective under optimal conditions, as demonstrated by the greatest increases in fresh and dry shoot weight as well as dry root weight when compared to the control. Moreover, all of the NP treatments that were assessed in drought-stressed conditions showed an increase in biomass accumulation, whereas, in comparison to the control, the NP Ultra treatment proved to be the most effective. In this regard, similar findings have been reported in soybean research, where it was found that Fe, Cu, Co, and ZnO NP may increase plant tolerance to drought stress by promoting the expression of drought-related genes, such as *GmWRKY27*, *GmMYB118*, and *GmMYB174*, involved in cell growth [83,84]. Cobalt is an essential nutrient for legumes since it is required for leghemoglobin synthesis and, thus, symbiotic nitrogen fixation. Seed coating with Co NPs enhances imbibition and germination rates as well as stand establishment, which could be helpful in reducing drought stress [85].

Also, nanopriming treatments had a favorable effect on the shoot elongation rate, where NP Ultra stood out as the most effective in both conditions. Contrary to this, in optimal conditions, NP Ultra reduced root elongation rate compared to the control. Moreover, the seedling vigor index was also enhanced due to nanopriming treatments compared to the control. According to the obtained results, a similar pattern was observed for previous parameters, where NP Ca-Si turned out to be more effective than other treatments in optimal conditions, while in drought-stressed conditions, it was NP Ultra. The same pattern was observed with SLSI and RLSI, parameters that more precisely determine the plant capacity for drought tolerance. Metal-based nanoprimings have proved to be excellent promoters of seed vigor and plant growth in stress conditions [86,87,88] due to their involvement in molecular responses against abiotic stresses, nutrient uptake and regulation, and the antioxidant defense system, which makes them a promising tool for improvement in sustainable agriculture.

Nevertheless, a poor migration of NPs in the soil might lead to their accumulatation and biomagnification [89]. Currently, there is a lack of information about the accumulation and toxicity of NPs in the soil. So far, it has been reported that some metal-based NPs are toxic to soil communities and plant-microbiome interactions [90]. The potentially adverse effects of NPs on biogeochemical cycles, such as nitrogen turnover and carbon emissions, have also been discussed [90]. However, seed nanopriming minimizes the exposure of the environment to NPs and is thus less harmful than the foliar use of NPs [91].

Additionally, it has been demonstrated that NPs that are accumulated in plant roots tend to translocate to other tissues, such as newly developed seeds and plant shoots, and that NP translocation is significantly influenced by the properties of both plants and the characteristics of NPs [92,93]. In this regard, it is reported that the accumulation of Fe and Zn could be increased in wheat and maize grains due to nanopriming [91,94,95]. However, the chemical mechanisms behind the uptake, absorption, and transport of nanoparticles in plants remain unclear [96,97]. Considering that a very small amount of nanoparticles is applied through seed priming, it is assumed that a greater accumulation of nanomaterials in the soil, as well as in the pea grain, cannot occur. The main difference with professional nanomaterials is that they provide high biological efficiency at hundreds of times lower consumption of microelements compared to traditional salt and chelate microfertilizers. Accordingly, the chemical load on the soil and plants is reduced. In the case of the priming operation, nanoparticles enter only the seeds and are almost completely used in the metabolic process as cofactors in the synthesis of metal-dependent enzyme proteins. The nanoparticles of microelements are synthesized in a cover of biogenic polymers, which are gradually absorbed by enzymes in plant cells with a dosed release of atoms of elements, leading to prolonged action and the absence of inhibition of plant development. It has been established that the bio-polymer-stabilized colloidal solutions of nanoparticles of microelements (Co, Mn, Cu, Fe, Zn, Cr, Se, Mo, and Ag) in various combinations and concentrations are low-toxic, non-eco-, and phytotoxic, and do not cause long-term consequences [98,99,100]. To the best of our knowledge, the phytotoxicity of these nanoprimings on pea grains and soil has not been examined. Thus, our future research will be focused on this field of study.

## 4. Materials and Methods

### 4.1. Nanoparticle (NPs)-Based Priming Materials

In this study, the effects of three different commercial nanomaterials used in crop production —Nanoplant Ultra (NP Ultra), Nanoplant Ca-Si (NP Ca-Si), and Nanoplant Sulphur (NP Sulphur) (JSC “ECO—Vlit”, Trakai, Lithuania)—on the seed quality of field pea under both optimal and drought-stressed conditions were assessed. All nanomaterials are developed at the National Academy of Sciences of Belarus. Organic polymers, such as polyvinpyrrolidone K17 and dextran D20, were used to encapsulate nanoparticles of elements to stabilize colloid structure.

Nanoplant Ultra is a commercial micronutrient product, which is complex nanomaterial-aqueous colloids, based on trace elements compounds: cobalt (Co)—0.036% (as CoFe_2_O_4_), manganese (Mn)—0.036% (as MnO_2_), copper (Cu)—0.043% (as CuS), iron (Fe)—0.06% (as Fe_2_O_3_), zinc (Zn)—0.025% (as ZnFe_2_O_4_), molybdenum (Mo)—0.045% (as MoO_2_), and selenium (Se)—0.045% (as Se0). NP Ultra nanopriming agent was prepared following the manufacturer’s instructions by adding 0.7 mL of NP Ultra product to 1 L of water.

Nanoplant Ca-Si is a commercial product—aqueous colloids, based on trace elements compounds: calcium (Ca)—0.5% (as Ca(BO_2_)_2_), silicon (Si)—0.05% (as Fe_2_SiO_4_), boron (B)—0.1% (as Ca(BO_2_)_2_, and iron (Fe)—0.1% (as Fe_2_SiO_4_). The nanopriming agent of NP Ca-Si was prepared following the manufacturer’s instructions by adding 2 mL of NP Ultra product to 1 L of water.

Nanoplant Sulfur is an aqueous colloidal commercial product containing nanoparticles of sulfur (S)-2.5% (as S). As prescribed by the manufacturer, the nanopriming agent was prepared by adding 1.5 mL to 1 L of water.

The pH of the prepared nanopriming agent was 7 ± 1, which corresponds to the pH of distilled water.

### 4.2. Characterization of Nanoparticles

Induced coupled plasma spectrometry (ICP, VISTA PRO (Varian, Palo Alto, CA, USA)) was used at the Institute of Physical Organic Chemistry, National Academy of Sciences of Belarus, Minsk, to measure the concentration of elements in the nanomaterial.

The traditional transmission electron microscopy method for measuring the size of nanoparticles implies a dry sample, while the water removal from volumetric nanoparticles leads to incorrect results that differ from the actual sizes of nanoparticles in the initial structure of aqueous colloids [101]. Therefore, dynamic scattering spectroscopy was used to measure the size of nanoparticles in the colloids [100,102] in two accredited laboratories: The Centre for Research and Testing of Materials of the Institute of Powder Metallurgy (Minsk, Belarus) and the Fraunhofer Institute for Ceramic Technologies and Systems IKTS (Dresden, Germany).

**Figure 2:**
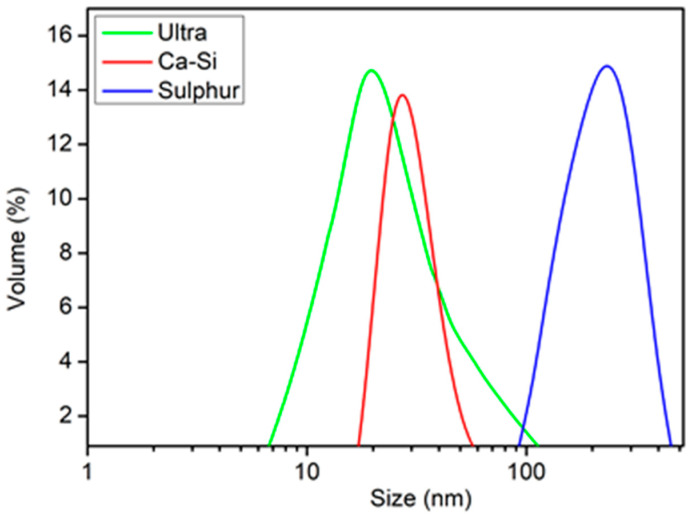
Diagram of nanoparticle size distribution in the three samples under study.

A laser analyzer based on the dynamic scattering method called “Zetasizer Nano ZSP” (Malvern, UK) was used to measure the granulometric composition of the colloids in both laboratories. The standard method for measuring colloids includes a measurement procedure with varying degrees of sample dilution with deionized water (from 1/10 to 1/1000) and additional processing of dilute colloidal solutions in an ultra-sonic field and a laboratory centrifuge. The measurement results are shown in the form of diagrams presenting the size distribution of nanoparticles for three studied samples characterized by high polydispersity (Figure 2). In the NP Ultra sample, the nanoparticles ranged in size from 6 to 106 nm (average size: 18–21 nm). In the NP Ca-Si sample, the nanoparticle size ranged from 16 to 68 nm (average size: 24–28 nm).

In the NP Sulfur sample, the particle size ranged from 90 to 450 nm (average size: 220–250 nm). This difference in the sizes of sulfur nanoparticles synthesized using a similar technology has been studied and described in detail [103]. Using the electron microscope method (SEM and TEM), it was established that the primary size of S nanoparticles is 50–55 nm. However, sulfur nanoparticles are hydrophobic and tend to aggregate. When measuring colloid sulfur using dynamic scattering spectroscopy using a Zetasizer Nano ZSP device (Malvern, UK), secondary aggregates of S with a size of more than 100 nm were identified. The results are consistent with the results presented in the study of Chaudhuri and Paria [103]. Manufacturers of Nanoplant Sulfur take into account this peculiarity of sulfur nanoparticles and produce the preparation in the form of a two-component solution with the recommendation of mixing the components on the day of application and using it to treat plants during the day.

### 4.3. Seed Material

Seeds of field pea (*Pisum sativum* L.) cultivar NS Junior were obtained from the Legume Department, Institute of Field and Vegetable Crops, National Institute of the Republic of Serbia, Novi Sad, Serbia. NS Junior is a spring, early cultivar that is intended for combined use as bulk fodder (forage) and for grain production. Its strong genetic potential has been demonstrated through a yield of 30–50 t ha^−1^ of green fodder and 2.5–5.0 t ha^−1^ of grains. This cultivar is known for being easy to harvest and having a crude protein level of 28% in the grain and 19–21% in the dry matter of the fodder. In Serbia, it is one of the most widely used cultivars of spring peas since it contains minimal anti-nutritive substances, such as lectins, tannins, and trypsin inhibitors, and does not require heat treatment of the grain [104].

The pea seeds were produced on a chernozem soil with a slightly alkaline pH reaction and a medium nutrient supply. The production site is located at the Rimski Šančevi (Vojvodina Province, Serbia) experimental field of IFVCNS (N 45°19′, E 19°50′) in 2022. An amount of 150 kg of mono-ammonium phosphate (MAP 12–52: 12% NH_4_, 52% P_2_O_5_) per hectare was applied before plowing.

### 4.4. Seed Priming

Field pea seeds were primed using the following treatments: hydropriming (seeds primed with distilled water) and three different nanopriming agents (Nanoplant Ultra, Nanoplant Ca-Si, and Nanoplant Sulfur). Non-primed seeds (without water and NPs) were used as the control. Pea seeds were fully immersed in priming agents (1:5 *w*/*v*) for 10 h at room temperature in the dark [23,42]. The seeds were then dried to a weight close to their initial weight after being thoroughly rinsed with distilled water, while the seed moisture content was 13.3%.

### 4.5. Laboratory Assay

To assess the effect of different nanopriming agents on seed quality and initial plant growth parameters of field peas in both optimal and drought-stressed conditions, the laboratory experiment was conducted at the Laboratory for Seed Testing, Institute of Field and Vegetable Crops, Novi Sad, Serbia. Thirty boxes in total, divided into two sets of working samples, were used in the laboratory experiment; fifteen boxes were used under optimal conditions, and fifteen were used under drought-stressed conditions. There were three replicates of the working sample, with 100 seeds in each. Primed and unprimed seeds were sown in 240 × 150 mm plastic boxes with double-layer filter paper. In the first set of samples, distilled water was used to moisten the filter paper for ideal conditions. The second set of samples’ filter papers was wetted using a solution of −0.49 MPa polyethylene glycol (PEG-6000) (Sigma Aldrich, St. Louis, MO, USA) to create artificial drought, which is the sensitivity threshold for germination and early plant growth for pea cultivars [105]. All samples were placed in the germination chamber at 20 °C with a day/night regime of 16/8 h for 8 days, according to ISTA [106]. The germination (first count) is defined as the percentage (%) of seeds in a given sample that germinated within a 5-day period and formed seedlings with well-developed essential structures [106]. The percentage of final germination and abnormal seedlings was identified 8 days after sowing [106].

### 4.6. Measurement of Growth-Related Parameters and Physiological Indexes

To obtain growth-related parameters, 25 seeds per replicate were rolled in filter paper moistened with distilled water for optimal conditions and with −0.49 MPa polyethylene glycol (PEG-6000) for drought-stressed conditions. Shoot length, root length, and fresh shoot and root weight of 8-day-old seedlings were determined by picking 10 normal seedlings per replicate. To obtain dry shoot and root weight, seedlings were oven-dried at 80 °C for 24 h [23,105].

Abdul-Baki and Anderson’s formula [107] was used to calculate the seedling vigor index (SVI):

SVI=FG×SL

where,

FG—final germination (%),
SL—Seedling length (cm).

Shoot elongation rate (SER) (mm day^−1^) and root elongation rate (RER) (mm day^−1^) were calculated using the formulas of Channaoui et al. [108]:

$$\mathrm{SER}=\frac{\mathrm{SLE}-\mathrm{SLS}}{T}$$

$$\mathrm{RER}=\frac{\mathrm{RLE}-\mathrm{RLS}}{T}$$

where,

SLS—Shoot length determined five days following seeding (mm),
SLE—Shoot length determined eight days following seeding (mm),
RLS—Root length determined five days following seeding (mm),
RLE—Root length determined eight days following seeding (mm),
T—The interval of time (in days) between two measurements (day).

Shoot length stress tolerance index (SLSI) and root length stress tolerance index (RLSI) were determined using the formulas of Bayat et al. [55]:

$$\mathrm{SLSI}=\frac{\mathrm{average}\;\mathrm{shoot}\;\mathrm{length}\;\mathrm{of}\;\mathrm{treated}\;\mathrm{seedlings}}{\mathrm{average}\;\mathrm{shoot}\;\mathrm{length}\;\mathrm{of}\;\mathrm{control}\;\mathrm{seedlings}\;}\times 100\;$$

$$\mathrm{RLSI}=\frac{\mathrm{average}\;\mathrm{root}\;\mathrm{length}\;\mathrm{of}\;\mathrm{treated}\;\mathrm{seedlings}}{\mathrm{average}\;\mathrm{root}\;\mathrm{length}\;\mathrm{of}\;\mathrm{control}\;\mathrm{seedlings}}\times 100$$

### 4.7. Statistical Analysis

The experiment was set up in a completely randomized design with three replications. The obtained data were statistically processed using the STATISTICA 10.0 (StatSoft Inc., Tulsa, OK, USA) software package. Analysis of variance (ANOVA), followed by mean separation according to Duncan’s multiple range test (p≤0.05), was performed. Pearson’s correlation analysis was performed to determine the relationship between the examined parameters.

## 5. Conclusions

In this study, the nanopriming treatments showed great potential for improving pea seed germination and initial plant growth in different conditions (optimal conditions and artificial drought). All examined treatments increased seed germination compared to the control, especially under drought. Additionally, nanopriming with the examined nanoparticles considerably increased initial plant growth and biomass accumulation. In terms of treatments, however, Nanoplant Ca-Si proved to be the most effective under optimal conditions, and Nanoplant Ultra was the most effective under drought conditions. It could be suggested that these priming nanomaterials can be used as a green strategy to improve seed quality and performance under different conditions. Future research should be conducted using three brands of nanomaterials when growing peas in the soil while modeling salinity conditions. Firstly, the seed quality, as well as important agronomic, physiological, and biochemical parameters, will be monitored under different environmental conditions in climate incubators and greenhouses. Additionally, the agronomic and production characteristics of peas will be evaluated in the field, with or without the application of fertilizers. Simultaneously, the phytotoxicity of three brands of nanomaterials and their impact on the soil will also be examined.

## Figures and Tables

**Table 1:** Analysis of variance (ANOVA) for parameters of field pea primed with different nanoparticles.

Traits	Factors		
	Condition (C)	Treatment (T)	C × T
Germination (First Count)	0.0000 ***	0.0000 ***	0.0000 ***
Final Germination	0.0000 ***	0.0000 ***	0.0037 ***
Abnormal Seedlings	0.0076 **	0.0054 **	0.0020 **
Shoot Length	0.0000 ***	0.0000 ***	0.0000 ***
Root Length	0.0000 ***	0.0000 ***	0.0001 ***
Fresh Shoot Weight	0.0000 ***	0.0000 ***	0.0022 ***
Fresh Root Weight	0.0000 ***	0.0000 ***	0.0000 ***
Dry Shoot Weight	0.0000 ***	0.0000 ***	0.0002 ***
Dry Root Weight	0.0000 ***	0.0000 ***	0.0000 ***
Shoot Elongation Rate	0.0027 **	0.0000 ***	0.0000 ***
Root Elongation Rate	0.0033 **	0.0001 ***	0.0030 **
Seedling Vigour Index	0.0000 ***	0.0001 ***	0.0016 **
Shoot Length Stress Tolerance Index	0.0105 *	0.0000 ***	0.0000 ***
Root Length Stress Tolerance Index	0.7405 ^ns^	0.0003 ***	0.0017 **

* p ≤0.05, ** p ≤0.01, *** p ≤0.001, ns—not significant; Condition (C) factor: optimal and drought stress conditions; Treatment (T) factor: control, hydropriming, and nanopriming (Nanoplant Ultra, Nanoplant Ca-Si, and Nanoplant Sulfur); C × T: interaction between factor condition and factor treatment.

**Table 2:** Effect of different seed nanopriming treatments on final germination and seedling length of field pea in (a) optimal conditions and (b) drought-stressed conditions.

Treatman	Germination (First Count) (%)	Final Germination (%)	Abnormal Seedlings (%)	Shoot Length (mm)	Root Length (mm)
	(a) Optimal Conditions				
Control	71.6 ± 0.67 c	78.7 ± 0.88 d	10.3 ± 0.88 a	105.5 ± 0.57 d	130.5 ± 2.18 b
HP	74.0 ± 0.58 b	79.7 ± 0.88 cd	5.0 ± 0.58 c	105.0 ± 1.04 d	130.7 ± 1.17 b
NP Ultra	74.3 ± 0.33 b	83.3 ± 0.67 ab	6.7 ± 0.33 bc	124.5 ± 0.29 b	133.5 ± 0.76 b
NP Ca-Si	77.0 ± 0.58 a	85.0 ± 0.58 a	9.0 ± 0.58 a	131.5 ± 1.53 a	144.2 ± 2.33 a
NP Sulfur	77.7 ± 0.88 a	81.3 ± 0.67 bc	8.3 ± 0.67 ab	112.7 ± 1.20 c	140.3 ± 0.73 a
	(b) Drought-stressed Conditions				
Control	53.7 ± 0.88 c	70.7 ± 0.67 c	7.3 ± 0.67 a	87.3 ± 0.60 d	107.0 ± 1.61 c
HP	65.0 ± 1.00 a	71.7 ± 0.88 c	7.0 ± 1.00 a	102.3 ± 0.44 b	110.5 ± 0.58 bc
NP Ultra	61.0 ± 1.15 b	75.3 ± 0.33 b	6.7 ± 0.67 a	109.5 ± 2.52 a	115.7 ± 1.76 a
NP Ca-Si	60.3 ± 0.88 b	76.7 ± 0.88 ab	5.0 ± 0.88 a	100.2 ± 0.67 b	110.5 ± 0.58 bc
NP Sulfur	52.7 ± 0.88 c	78.7 ± 0.67 a	7.0 ± 0.58 a	92.3 ± 0.60 c	111.7 ± 0.33 b

* Data are represented as mean ± SE (*n* = 3; 100 seeds per replicate for germination first count, final germination, and abnormal seedlings; 10 seedlings per replicate for shoot and root length). Differences between priming treatments were analyzed using Duncan’s multiple range test (*p* ≤ 0.05). The means ± SE for each trait, denoted by the different letters, differ significantly. Control, unprimed seeds; HP, hydropriming; NP Ultra, Nanoplant ultra; NP Ca-Si, Nanoplant Ca-Si; NP Sulfur, Nanoplant Sulfur.

**Table 3:** Effect of different seed nanopriming treatments on biomass accumulation of filed pea in (a) optimal conditions and (b) drought-stressed conditions.

Treatment	Fresh Shoot Weight (g)	Fresh Root Weight (g)	Dry Shoot Weight (g)	Dry Root Weight (g)
	(a) Optimal Conditions			
Control	2.70 ± 0.01 c	1.52 ± 0.01 c	0.231 ± 0.003 c	0.128 ± 0.004 c
HP	2.68 ± 0.01 c	1.59 ± 0.03 c	0.238 ± 0.003 c	0.132 ± 0.000 c
NP Ultra	3.15 ± 0.01 ab	1.59 ± 0.01 c	0.254 ± 0.003 b	0.187 ± 0.002 b
NP Ca-Si	3.25 ± 0.06 a	1.96 ± 0.04 b	0.267 ± 0.002 a	0.209 ± 0.004 a
NP Sulfur	3.10 ± 0.04 b	2.10 ± 0.06 a	0.267 ± 0.002 a	0.133 ± 0.001 c
	(b) Drought-stressed Conditions			
Control	2.10 ± 0.03 c	1.36 ± 0.01 d	0.181 ± 0.001 b	0.123 ± 0.000 c
HP	2.14 ± 0.03 c	1.43 ± 0.02 c	0.179 ± 0.005 b	0.122 ± 0.001 c
NP Ultra	2.46 ± 0.04 a	1.57 ± 0.01 a	0.210 ± 0.002 a	0.142 ± 0.004 a
NP Ca-Si	2.52 ± 0.01 a	1.50 ± 0.02 b	0.183 ± 0.007 b	0.128 ± 0.001 bc
NP Sulfur	2.28 ± 0.02 b	1.44 ± 0.01 c	0.198 ± 0.004 a	0.134 ± 0.003 b

* Data are represented as mean ± SE (*n* = 3; 10 seedlings per replicate). Differences between priming treatments were analyzed using Duncan’s multiple range test (*p* ≤ 0.05). The means ± SE for each trait, denoted by the different letters, differ significantly. Control, unprimed seeds; HP, hydropriming; NP Ultra, Nanoplant ultra; NP Ca-Si, Nanoplant Ca-Si; NP Sulfur, Nanoplant Sulfur.

**Table 4:** Effect of different seed nanopriming treatments on shoot and root elongation rate, seedling vigor index, and stress tolerance index of filed pea in (a) optimal conditions and (b) drought-stressed conditions.

Treatment	Shoot Elongation Rate (mm day^−1^)	Root Elongation Rate (mm day^−1^)	Seedling Vigor Index	SLSI (%)	RLSI (%)
	(a) Optimal Conditions				
Control	22.32 ± 0.31 c	14.94 ± 0.61 a	1879.1 ± 39.1 c	100.0 ± 0.00 d	100.0 ± 0.00 c
HP	22.11 ± 0.40 c	16.61 ± 0.45 a	2029.2 ± 63.3 bc	99.54 ± 1.52 d	100.1 ± 0.93 c
NP Ultra	28.33 ± 0.10 a	11.61 ± 0.28 b	1962.8 ± 54.2 bc	118.02 ± 0.56 b	102.3 ± 1.36 bc
NP Ca-Si	28.39 ± 0.70 a	15.72 ± 0.82 a	2342.5 ± 45.0 a	124.66 ± 2.00 a	110.6 ± 3.07 a
NP Sulfur	24.33 ± 0.35 b	15.50 ± 0.19 a	2057.8 ± 22.9 b	106.8 ± 1.39 c	107.6 ± 1.60 ab
	(b) Drought-stressed Conditions				
Control	21.17 ± 0.25 d	13.28 ± 0.63 a	1480.0 ± 57.38 bc	100.0 ± 0.00 d	100.0 ± 0.00 c
HP	25.39 ± 0.31 b	14.28 ± 0.44 a	1418.9 ± 66.89 c	117.1 ± 0.75 b	103.3 ± 1.43 bc
NP Ultra	27.50 ± 0.92 a	13.44 ± 0.44 a	1696.2 ± 39.29 a	125.4 ± 3.68 a	108.1 ± 0.06 a
NP Ca-Si	23.56 ± 0.29 c	13.61 ± 0.39 a	1614.1 ± 24.31 ab	114.7 ± 1.43 b	103.3 ± 1.43 bc
NP Sulfur	22.89 ± 0.40 c	14.50 ± 0.44 a	1605.2 ± 65.96 ab	105.7 ± 0.039 c	104.4 ± 1.33 b

* Data are represented as mean ± SE (*n* = 3; 10 seedlings per replicate). Differences between priming treatments were analyzed using Duncan’s multiple range test (p≤0.05). The means ± SE for each trait, denoted by the different letters, differ significantly. Control, unprimed seeds; HP, hydropriming; NP Ultra, Nanoplant ultra; NP Ca-Si, Nanoplant Ca-Si; NP Sulfur, Nanoplant Sulfur. SLSI, Shoot Length Stress Tolerance Index; RLSI, Root Length Stress Tolerance Index.

**Table 5:** Correlation analysis of germination, initial plant growth, and stress tolerance indexes of a field pea.

(a) Optimal Conditions														
Variable	GFC	FG	AS	SL	RL	FSW	FRW	DSW	DRW	SER	RER	SVI	SLSI	RLSI
GFC	1.00	0.65 **	−0.04 ^ns^	0.47 ^ns^	0.74 **	0.74 **	0.87 ***	0.89 ***	0.37 ^ns^	0.40 ^ns^	0.13 ^ns^	0.63 *	0.45 ^ns^	0.79 ***
FG		1.00	−0.02 ^ns^	0.87 ***	0.61 *	0.86 ***	0.45 ^ns^	0.74 ***	0.85 ***	0.84 ***	−0.30 ^ns^	0.54 *	0.84 ***	0.72 **
AS			1.00	0.07 ^ns^	0.29 ^ns^	0.13 ^ns^	0.23 ^ns^	0.09 ^ns^	0.03 ^ns^	−0.01 ^ns^	0.02 ^ns^	0.05 ^ns^	0.08 ^ns^	0.22 ^ns^
SL				1.00	0.66 **	0.87 ***	0.38 ^ns^	0.70 **	0.96 ***	0.98 ***	−0.36 ^ns^	0.64 *	0.99 ***	0.60 *
RL					1.00	0.79 ***	0.83 ***	0.84 ***	0.52*	0.55 *	0.29 ^ns^	0.74 **	0.66 **	0.87 ***
FSW						1.00	0.66 **	0.88 ***	0.77 ***	0.85 ***	−0.31 ^ns^	0.59 **	0.86 ***	0.76 ***
FRW							1.00	0.84 ***	0.21 ^ns^	0.28 ^ns^	0.29 ^ns^	0.60*	0.37 ^ns^	0.77 ***
DSW								1.00	0.57*	0.65 **	−0.05 ^ns^	0.62*	0.69 **	0.81 ***
DRW									1.00	0.92 ***	−0.38 ^ns^	0.65 **	0.95 ***	0.49 ^ns^
SER										1.00	−0.51 ^ns^	0.49 ^ns^	0.98 ***	0.48 ^ns^
RER											1.00	0.31 ^ns^	−0.35 ^ns^	0.22 ^ns^
SVI												1.00	0.65 **	0.59 *
SLSI													1.00	0.56 **
RLSI														1.0000
(b) Drought-stressed Conditions														
GFC	1.00	−0.25 ^ns^	−0.27 ^ns^	0.752 ***	0.21 ^ns^	0.20 ^ns^	0.44 ^ns^	−0.10 ^ns^	−0.09 ^ns^	0.66 **	−0.11 ^ns^	−0.15 ^ns^	0.75 ***	0.28 ^ns^
FG		1.00	−0.25 ^ns^	0.16 ^ns^	0.51 ^ns^	0.64 **	0.44 ^ns^	0.49 ^ns^	0.60 *	0.13 ^ns^	0.40 ^ns^	0.66 **	0.14 ^ns^	0.43 ^ns^
AS			1.00	−0.22 ^ns^	−0.17 ^ns^	−0.49 ^ns^	−0.43 ^ns^	0.04 ^ns^	−0.08 ^ns^	−0.03 ^ns^	0.05 ^ns^	−0.25 ^ns^	−0.19 ^ns^	−0.10 ^ns^
SL				1.00	0.72 **	0.59 *	0.83 ***	0.37 ^ns^	0.44 ^ns^	0.96 ***	0.03 ^ns^	0.38 ^ns^	0.99 ***	0.70 **
RL					1.00	0.56 *	0.78 ***	0.68 **	0.71 **	0.76 ***	0.40 ^ns^	0.63 *	0.69 **	0.73 **
FSW						1.00	0.83 ***	0.46 ^ns^	0.54 *	0.44 ^ns^	−0.06 ^ns^	0.65 **	0.58 *	0.56 *
FRW							1.00	0.56 *	0.64 **	0.75 ***	−0.06 ^ns^	0.68 **	0.83 ***	0.75 ***
DSW								1.00	0.81 ***	0.41 ^ns^	0.09 ^ns^	0.52 *	0.34 ^ns^	0.77 ***
DRW									1.00	0.47 ^ns^	−0.00 ^ns^	0.66 **	0.40 ^ns^	0.70 **
SER										1.00	0.09 ^ns^	0.37 ^ns^	0.96 ***	0.69 **
RER											1.00	0.13 ^ns^	−0.03 ^ns^	0.12 ^ns^
SVI												1.00	0.37 ^ns^	0.61 **
SLSI													1.00	0.68 **
RLSI														1.0000

* p ≤0.05, ** p ≤0.01, *** p ≤0.001, ns—not significant. GFC, germination (first count); FG, final germination; AS, abnormal seedlings; SL, shoot length; RL, root length; FSW, fresh shoot weight; FRW, fresh root weight; DSW, dry shoot weight; DRW, dry root weight; SER, shoot elongation rate; RER, root elongation rate; SVI, seedling vigor index; SLSI, shoot length stress tolerance index; RLSI, root length stress tolerance index.

## Data Availability

The datasets used and analyzed during the current study are available from the corresponding author upon reasonable request.
